# Unlocking Team Potential: Mastering Communication in Palliative Care

**DOI:** 10.7759/cureus.74417

**Published:** 2024-11-25

**Authors:** Paula Cerqueira, Sara Pereira, Raquel Costa, Bárbara Sousa

**Affiliations:** 1 Internal Medicine - Medicina 2, Unidade Local de Saúde do Alto Minho - Hospital Conde de Bertiandos, Ponte de Lima, PRT; 2 Internal Medicine, Hospital Distrital de Santarém, Santarém, PRT

**Keywords:** communication, compassionate care, holistic evaluation, patient-centered care multidisciplinary approach, technology

## Abstract

Effective communication is crucial in multidisciplinary teams (MDTs) within palliative care, where patient needs can be complex and multifaceted. This article examines the significance of communication in promoting collaborative, patient-centered care while addressing challenges such as professional jargon, hierarchical barriers, and the emotional strain associated with end-of-life care. Leadership plays a vital role in creating an environment of open dialogue, reducing hierarchical dynamics, facilitating conflict resolution, and supporting the emotional well-being of team members. The article also outlines strategies to improve communication, including regular team meetings, the use of clear and inclusive language, communication skills training, and the integration of technology. By implementing these strategies, MDTs can overcome communication barriers, leading to better patient outcomes, enhanced team cohesion, and improved family satisfaction. In palliative settings, effective communication is essential for providing holistic and compassionate care.

## Introduction and background

The complexity of patient needs in palliative care calls for communication practices that address the multifaceted dimensions of suffering. Saunders’ “total pain” concept underscores the importance of viewing suffering holistically, encompassing physical pain and emotional, social, and spiritual distress [1]. This perspective establishes a foundational need for open, interdisciplinary communication to ensure no aspect of a patient’s experience is neglected. Similarly, Gawande highlighted how understanding and aligning care with patient priorities through meaningful conversations is central to patient-centered care [2]. Both emphasize that effective communication is a cornerstone of holistic palliative care. Therefore, effective communication is the cornerstone of successful palliative care, where multidisciplinary teams (MDTs) work together to provide holistic care to patients with life-limiting illnesses [3,4]. Palliative care MDTs typically include physicians, nurses, social workers, chaplains, and other healthcare professionals, each bringing their expertise to address patients' and their families' complex physical, emotional, social, and spiritual needs [5]. In such a setting, communication is not just a tool for exchanging information; it is essential for ensuring that the team functions cohesively, that patient care is coordinated and personalized, and that the patient and family's preferences are respected [6].

The importance of communication in MDTs 

Communication is the cornerstone of effective teamwork in MDTs, particularly in palliative care, where patient needs are complex and require input from diverse professionals, including doctors, nurses, social workers, spiritual care providers, and psychologists [7]. Effective communication ensures that each team member's expertise is integrated into comprehensive, patient-centered care, directly impacting patient outcomes, family satisfaction, and team dynamics [6,7]. Given the intricacies of end-of-life care, where decisions are often sensitive and emotional, the quality of communication can directly impact patient outcomes, family satisfaction, and team dynamics [6,7]. One primary reason communication is vital in MDTs is its role in facilitating collaborative care [8]. Each team member brings a unique perspective based on their professional background and area of expertise. Physicians may focus on the medical aspects of care, such as pain management and symptom control, while nurses might concentrate on patient comfort and practical daily care [7]. Social workers address psychosocial concerns, and spiritual advisors offer guidance and support in spiritual matters, whether or not they are tied to a specific religion [7]. Effective communication bridges these silos, ensuring that every aspect of the patient’s care plan is discussed and integrated [9]. Through regular team meetings, case conferences, and informal discussions, MDT members can share their observations and recommendations, leading to a more comprehensive understanding of the patient's needs [10]. By fostering an open flow of information, communication allows MDTs to deliver seamless, coordinated care that reflects the patient's medical, emotional, and spiritual needs [7]. 

In palliative care, the patient's quality of life is the central focus, and communication within MDTs is crucial in ensuring that care remains patient and family-centered [3]. Given that patients in palliative care often face complex medical and emotional challenges, effective communication within the team is essential for aligning care with the patient's goals, preferences, and values [11]. With clear and consistent communication, the team can collectively develop a care plan that reflects the patient's wishes. For instance, a patient's decision about pursuing aggressive treatment or focusing on comfort care may require input from various professionals. A nurse might observe changes in the patient's physical condition, while a psychologist could provide insights into the patient’s emotional readiness for certain decisions. Social workers and chaplains may offer perspectives on the patient's values and family dynamics [7]. These different viewpoints can be synthesized through open communication to create a care plan that respects the patient's dignity and autonomy [5,7]. Moreover, communication among team members is essential for ensuring that the patient's family is adequately supported throughout the care process. Families of palliative care patients often face difficult decisions and emotional challenges, and they rely on the care team for guidance and reassurance. When the MDT communicates effectively, it can present a unified message to the family, avoiding confusion or conflicting information. This consistency helps build family trust, reduces anxiety, and ensures they feel involved in decision-making [11]. 

Communication breakdowns are a common cause of medical errors in healthcare settings, and MDTs in palliative care are no exception [12]. Palliative care often involves managing complex medication regimens, coordinating care across different settings (e.g., hospitals, hospices, and home care), and responding to rapidly changing patient conditions. In such a dynamic environment, communication is essential to prevent errors and ensure continuity of care [12]. For example, a lapse in communication during handovers between shifts or when a patient is transferred between care settings can result in missed information, such as changes in medication or treatment goals. Such miscommunications can have serious consequences, including unnecessary treatments, unmanaged symptoms, or not following the patient's will and the goal of comfort [13]. To mitigate this risk, MDTs rely on structured communication institutional protocols, such as standardized handover tools and interdisciplinary rounds, which help ensure that critical information is consistently shared among all team members [13]. Additionally, communication plays a crucial role in updating the care plan as the patient's condition evolves. The team must be informed of new developments in palliative care, where patient needs can change rapidly. For example, suppose a patient's condition deteriorates, and the decision is made to shift care focus from curative treatment to comfort measures. In that case, every member of the MDT must be aware of this change. Effective communication ensures that all team members are aligned with the updated care goals and can adjust their interventions accordingly, maintaining a high patient care standard [14].

Finally, communication is essential for building trust and fostering team cohesion in MDTs. Open and transparent communication promotes mutual respect and trust among team members, enabling them to collaborate effectively. In palliative care, where decisions are often time-sensitive and high-pressure, trust ensures that team members feel confident sharing their insights and perspectives [7,8]. Trust strengthens team dynamics, encouraging members to share information freely and support one another in their roles, ultimately leading to more cohesive and coordinated care [10]. Leadership is vital in cultivating this environment by fostering openness and inclusivity. Influential leaders actively encourage contributions from all team members, regardless of rank or discipline, ensuring diverse perspectives are integrated into decision-making [7,13]. Inclusive leadership practices improve communication and boost team morale and job satisfaction. By breaking down hierarchical barriers, leaders create a culture where critical insights and concerns are openly shared and addressed, enhancing the team's ability to deliver high-quality, patient-centered care [8].

## Review

Despite the critical role communication plays in MDTs within palliative care, it is fraught with numerous challenges that can impede the delivery of comprehensive care [14]. These challenges stem from various factors, including differences in professional language, experience, and expertise among team members and the profound emotional burden of caring for patients at the end of life [14,15]. The complexity inherent in palliative care, where patient needs are multifaceted and constantly evolving, necessitates ongoing coordination and seamless information sharing. However, maintaining this level of communication in a high-pressure environment is often difficult, leading to potential breakdowns in teamwork and patient care [16].

Challenges in communication in multidisciplinary palliative care teams

One of the most significant challenges in MDT communication arises from professional jargon and discipline-specific language. Each healthcare discipline within the team, whether medicine, nursing, social work, or spiritual advisors, has specialized terminology [9]. While this jargon is efficient within individual disciplines, it can create substantial barriers when communicating across different fields [10]. This can result in miscommunication, where critical information is either lost in translation or misinterpreted, potentially leading to suboptimal patient care or even clinical errors [12]. The challenge of language barriers is not just limited to jargon but also extends to how professionals from various backgrounds communicate. For example, a physician might communicate information clinically and concisely, focusing on medical facts and prognosis, while a social worker might focus on psychosocial aspects, using more empathetic language. When these communication styles are aligned, or efforts are made to bridge the differences between these professionals, care becomes more cohesive, ensuring the patient and family receive clear and comprehensive information instead of mixed messages [17]. 

Another significant challenge in MDT communication is the presence of hierarchical structures within healthcare teams. Traditional medical environments often have a clearly defined hierarchy, with physicians typically at the top, followed by nurses, allied health professionals, and support staff [8]. While this hierarchy can streamline decision-making, it can also stifle open communication, particularly from junior team members or those in less authoritative roles [8]. For example, a junior nurse or a social worker might hesitate to voice a concern or offer a differing opinion during a case discussion, especially if it contradicts the view of a more senior physician. This can lead to critical insights being overlooked, and the patient's care plan may only partially reflect the collective expertise of the entire team [14]. Moreover, this hierarchical communication dynamic can foster an environment where less senior team members feel undervalued, leading to disengagement and reduced collaboration. Hierarchical challenges are further exacerbated in multicultural teams, where cultural perceptions of authority and communication may differ. In some environments, questioning a senior colleague or openly disagreeing in a public forum may be considered disrespectful, limiting the exchange of ideas and the quality of team discussions [15].

The emotional and ethical dimensions of palliative care introduce additional layers of complexity to MDT communication. Palliative care involves discussions around mortality, suffering, and the moral dilemmas associated with end-of-life decisions [15]. These sensible topics require clear communication and a high degree of empathy, emotional intelligence, and ethical awareness. Rothweiler et al.'s work illustrates how denial or anger in patients and families can complicate communication, particularly if teams fail to approach these emotions sensitively [18]. Healthcare professionals working in palliative care often experience emotional stress as they navigate the complexities of patient suffering, family dynamics, and their responses to death and dying [17]. This emotional burden can sometimes hinder effective communication, as team members might avoid difficult conversations, downplay their emotions, or become overly clinical to manage their stress [15]. Puchalski et al.’s emphasis on spirituality adds another layer, as MDTs often struggle to discuss or integrate spiritual concerns due to a lack of training or discomfort in broaching such topics [19].

Moreover, ethical challenges often arise when there is a divergence of opinions within the team about the best course of action for a patient. For instance, there may be disagreements about the continuation of aggressive treatment versus transitioning to comfort care, especially when there are no clear directives from the patient or family. These situations require careful, respectful communication where all voices are heard, and the team collectively must navigate the ethical complexities [17]. Failure to manage these discussions effectively can lead to conflicts within the team, moral distress among healthcare providers, and, ultimately, compromised patient care [20].

Strategies for effective communication in multidisciplinary palliative care teams

Various strategies can be employed to overcome the communication challenges that MDTs face in palliative care. These strategies foster a more cohesive and effective team environment where communication flows freely and accurately, ensuring that patient care is comprehensive and coordinated [21].

One of the most effective strategies to enhance communication within palliative care MDTs is the implementation of regular team meetings and debriefings. These structured meetings provide a dedicated forum for team members to discuss patient cases in detail, share pertinent information, and align their care strategies [22]. During these sessions, team members should address any issues or uncertainties regarding a patient’s care plan, ensuring that all perspectives are considered and the team works towards common goals. Debriefings, particularly following critical incidents or after the death of a patient, are equally important. These sessions allow the team to reflect on the care provided, process their emotional responses, and learn from the experiences. Such reflections can improve future care and help team members cope with the emotional demands of palliative care [8]. Regular debriefings also build team cohesion, providing a safe space for open dialogue and mutual support among team members [8]. Implementing structured interdisciplinary rounds and standardized handover protocols is another effective strategy for enhancing communication. Interdisciplinary rounds provide a regular opportunity for all team members to come together and discuss the care of patients in a coordinated manner. During these rounds, each discipline can present its perspective on the patient’s care, ensuring that all aspects of the patient’s needs are addressed [23]. Standardized handover protocols are equally crucial in providing continuity of care. These protocols ensure that critical information is consistently shared across the team during transitions, such as shift changes or when a patient is transferred from one care setting to another. Following a standardized process dramatically reduces the risk of information loss or miscommunication, which is crucial in the complex palliative care environment [23].

Another critical strategy for improving communication within MDTs is transparent and inclusive language. Given the diverse professional backgrounds of team members, it is essential to use language accessible to all disciplines. This means avoiding jargon or, when it is necessary, taking the time to explain any technical terms [12]. When team members communicate clearly and understandably, it reduces the risk of misinterpretations. It ensures that everyone has a shared understanding of the patient’s condition and care plan. Inclusive language also plays a role in fostering a respectful and collaborative environment. When team members feel that the communication is tailored to their understanding and that their contributions are valued, they are more likely to engage actively in discussions. This inclusive approach helps bring out the best in each team member, ensuring that the care provided is genuinely multidisciplinary [16].

Leadership plays a crucial role in modeling openness and inclusivity [24]. Leaders should encourage all team members to contribute to discussions, regardless of rank or discipline, ensuring that a wide range of perspectives are considered. This can be achieved by actively soliciting input from less senior members during meetings and clarifying that all contributions are valued [24]. Such a culture improves communication and enhances team morale and job satisfaction. When team members feel that their opinions are respected and part of a collaborative effort, they are more likely to fully engage with the team’s goals and provide the best possible patient care [8]. Moreover, an inclusive culture helps to break down hierarchical barriers that can stifle communication, ensuring that essential insights and concerns are not overlooked [24].

Ongoing training in communication skills is another essential strategy for enhancing MDT effectiveness. Training sessions can cover various topics, including active listening, empathy, conflict resolution, and delivering difficult news [25]. Such training empowers team members with the skills they need to communicate effectively with each other and with patients and families. For instance, active listening is crucial in ensuring that all team members feel heard and that their contributions are fully understood. Empathy is essential in palliative care, not just in patient interactions but also in understanding fellow team members' emotional states and perspectives [24]. Conflict resolution skills are critical in MDTs, where differing opinions can sometimes lead to tension. Training in these areas helps prevent and manage conflicts, ensuring that the team remains focused on delivering patient-centered care [24].

Finally, the use of technology can significantly enhance communication within MDTs. Digital tools such as electronic health records (EHRs) and communication platforms support real-time information sharing and documentation [26]. These tools ensure that all team members can access the latest patient information, regardless of where or when they last interacted with the patient. For example, EHRs can be used to document patient preferences, clinical notes, and care plans in a way that is accessible to all members of the MDT. Communication platforms can facilitate instant messaging or video conferencing, enabling team members to discuss patient care even when they are not physically present in the exact location. Their integration is increasingly seen as a standard practice to overcome traditional communication barriers. By leveraging these technologies, teams can enhance their communication efficiency, reduce the likelihood of errors, and improve overall patient care [26].

The role of leadership in communication in multidisciplinary palliative care teams

Leadership is pivotal in shaping the communication dynamics within MDTs in palliative care. Effective leadership is about guiding the team in clinical decision-making and fostering an environment where open, transparent, and respectful communication is the norm [23]. One of the primary responsibilities of leaders in palliative care MDTs is to set the tone for open and inclusive communication. Leaders must actively encourage all team members, regardless of their rank or discipline, to contribute to discussions [27]. This can be achieved by modeling openness leaders who regularly seek input from others, admit uncertainties, and are willing to listen without judgment to create a safe space where team members feel comfortable sharing their perspectives [26]. For instance, during team meetings, a leader might explicitly invite input from quieter members or those from disciplines that are typically less vocal in medical discussions, such as social work or spiritual advisors. By doing so, leaders demonstrate that every contribution is valued, which is crucial in a setting where diverse perspectives are necessary to address the complex needs of palliative care patients [27]. When team members feel that their opinions are respected and considered, they are more likely to engage actively, leading to more thorough and holistic patient care [27].

Leadership in palliative care MDTs also involves addressing and mitigating the effects of hierarchical barriers that can impede communication. In many healthcare settings, traditional hierarchies can create an environment where junior team members or those from non-medical disciplines might feel reluctant to speak up, mainly if their views differ from those of more senior or medically authoritative colleagues [23]. This reluctance can lead to overlooking important insights and stifle the collaborative spirit essential in palliative care. Influential leaders work to flatten these hierarchies, at least in team communication, by promoting a culture of equality during discussions [27]. Leaders can also establish protocols that ensure each team member has an opportunity to speak, such as round-robin discussions or structured feedback sessions. By actively dismantling hierarchical barriers, leaders help create a more inclusive environment where all team members feel empowered to contribute [23].

Conflict is inevitable in any team setting, particularly palliative care, where decisions are often complex and emotionally charged. Leaders are critical in facilitating conflict resolution within the MDT, ensuring that disagreements are handled constructively and do not detract from patient care [27]. Influential leaders are skilled in recognizing the early signs of conflict and intervening before tensions escalate. They understand that conflict, when managed well, can lead to more robust decision-making as it brings different viewpoints to the forefront. Leaders can facilitate conflict resolution by promoting open dialogue and encouraging team members to openly express their concerns and disagreements. When conflicts arise, a leader might convene a special meeting to allow team members to voice their differing opinions in a controlled and respectful environment.

Leadership in palliative care also involves providing emotional support to the MDT and recognizing the emotional toll that end-of-life care can take on healthcare professionals. Palliative care is inherently challenging, with team members regularly facing the suffering and death of patients. Leaders must be attuned to the emotional states of their team members and provide the support necessary to maintain their psychological well-being and resilience [27]. This can involve creating opportunities for team members to express their emotions and share their experiences, such as during debriefings after the death of a patient or in regular support group meetings. Leaders might also advocate for access to professional counseling services for team members struggling with their work's emotional demands [23]. By prioritizing the team's emotional well-being, leaders help prevent burnout and ensure that team members can continue to provide compassionate care to their patients. Moreover, leaders play a crucial role in normalizing the expression of emotions within the team. There is an unspoken expectation in many healthcare environments that professionals should remain emotionally detached. However, in palliative care, where the work is intensely personal and emotionally charged, this detachment is neither realistic nor healthy [23]. Leaders who openly acknowledge their emotional responses and encourage others to do the same help create a more supportive and empathetic team environment. This enhances team cohesion and improves the overall quality of care, as emotionally healthy team members can better engage with their patients and their families on a deeper level [27].

Finally, effective leadership in palliative care MDTs involves fostering a culture of continuous learning and improvement. Communication is a skill that can constantly be refined, and leaders must be committed to developing their team’s communication abilities [28]. This can be achieved through regular training sessions on communication skills, such as active listening, empathy, and delivering difficult news, which are essential in palliative care [24]. Leaders can also promote a culture of feedback, where team members regularly reflect on their communication practices and seek ways to improve. For instance, after a family meeting or a particularly challenging case, a leader might initiate a debriefing focused on how well the team communicated and what could be done differently in the future [23]. By encouraging this reflective practice, leaders help their teams continuously enhance their communication skills. In addition to formal training and feedback, leaders can foster learning by staying informed about the latest developments in palliative care and communication practices and sharing this knowledge with their teams. This might involve bringing in external experts for workshops, encouraging team members to attend relevant conferences, or facilitating discussions on new research articles [23]. By prioritizing continuous learning, leaders ensure that their teams remain at the forefront of best practices in palliative care communication [24].

## Conclusions

Effective communication is fundamental to providing high-quality care in multidisciplinary palliative care teams. While communication challenges are common, they can be addressed through structured practices, a supportive team culture, and strong leadership. Prioritizing communication enhances service delivery, enabling teams to offer comprehensive, compassionate, and well-coordinated care. However, while the structure and processes of MDT communication have evolved, challenges remain. Critical areas for improvement include addressing hierarchical barriers, ensuring equitable participation, and maximizing the efficiency of MDTMs to balance administrative tasks with patient engagement. Future research should focus on evaluating the long-term impact of communication innovations on patient care and team dynamics. This approach ensures that the complex needs of patients and their families are effectively met, fostering trust and improving the overall care experience.
